# 机器人肿瘤手术的远期疗效值得期待

**DOI:** 10.3779/j.issn.1009-3419.2018.03.20

**Published:** 2018-03-20

**Authors:** 群友 谭

**Affiliations:** 第三军医大学大坪医院胸外科 Department of Thoracic Surgery, Daping Hospital, The Third Military Medical University

根据目前美国国家综合癌症网络（National Comprehensive Cancer Network, NCCN）指南的推荐意见，胸腔镜非小细胞肺癌根治术相比传统开放手术具有多方面的优点，如术后疼痛减轻，缩短住院时间，减少术后并发症的发生和提高预后等。随着机器人手术的出现及发展，达芬奇机器人辅助肺癌根治术逐渐应用于临床。但是其近远期疗效仍有较大争议。正是基于此，该研究对达芬奇机器人与胸腔镜辅助非小细胞肺癌根治术的近期疗效进行回顾性对比分析，作者认为，"达芬奇机器人行非小细胞肺癌根治术安全、有效，在近期疗效上优于胸腔镜组"。

在阅读全文后，我想针对该研究谈几点自己的看法：

第一、在国内开展机器人胸外科手术的单位不多、部分术者对其给患者带来的益处尚有疑虑的情况下，该研究结果令人鼓舞。

第二、该研究为配对的病例对照研究，为一种回顾性的临床研究。研究者对纳入研究的病例组和对照组的一般信息进行了匹配，以求尽可能减少偏倚的影响，研究结论比较可靠。当然，我们知道，具有最高证据等级的临床研究为前瞻性随机对照研究，该研究只能说明达芬奇机器人手术与更好的近期疗效相关，确切的结论尚需更进一步的前瞻性研究。

第三、该研究比较了两组的手术时间、术中失血量、淋巴结清扫数量、淋巴结清扫站数、术后带管时间、术后住院天数、术后第1日胸引液的量以及术后胸引液的总量等，应该说包括了绝大多数反映手术近期疗效的内容。但该研究并未对一些重要的研究参数进行准确的定义。如手术时间，在不同文献中的定义不同：有的定义为从进入手术室开始至出手术室止；另一些文献定义为从麻醉开始至皮肤缝合结束；还有的定义为从切皮开始至皮肤缝合结束。我们知道，机器人手术的准备程序较传统的手术要繁杂，这势必可能耽搁更多的时间，但由于机器人手术的精准操作，只要术者熟练掌握操作技术，完全可能缩短胸腔内操作时间。因此，若能准确定义研究参数，有利于读者理解以及减少分歧。

第四，在术后近期疗效评价方面，该研究并未涉及术后并发症的情况。而围术期并发症包括呼吸系统并发症、心血管系统并发症、疼痛等级等等对于患者术后恢复至关重要，若能进一步评价两者的术后并发症情况，则能更全面的支持作者的结论。

虽然机器人手术逐渐成为热点，但是由于手术机器人设备配置和费用等方面的限制，其在临床中的应用仍未达到像胸腔镜手术那样普及。目前针对机器人手术的临床研究仍较为缺乏，因此，该研究仍有一定的借鉴意义。该研究数据支持达芬奇机器人手术与更好的术后近期疗效相关，为进一步的临床研究提供了一定的基础。相信随着术者、患者对机器人手术的逐渐接受，手术机器人的优化升级，加之设备和手术费用的有效控制，机器人手术会越来越普及，与胸腔镜手术一道，成为我们外科医生的好帮手，服务于广大患者朋友。

